# Are narcissists always bad apples? The relationship between employee narcissism and creative deviance

**DOI:** 10.3389/fpsyg.2022.1026649

**Published:** 2022-11-17

**Authors:** Kaixin Zhang, Zilong Cui

**Affiliations:** ^1^Department of Business Administration, School of Manangement, Changchun Guanghua University, Changchun, Jilin, China; ^2^Department of Human Resource Management, Yatai College of Business Administration, Jilin University of Finance and Economics, Changchun, China

**Keywords:** narcissism, creative self-efficacy, creative deviance, transformational leadership, creativity

## Abstract

This study aims to advance the understanding of the effect of employee narcissism on creative deviance through creative self-efficacy and the moderation of this effect through transformational leadership. Research data were collected using a three-wave lagged model (*n* = 446) from 446 employees of 6 Chinese companies to test our moderated mediation model. The findings show that narcissism positively and significantly predicted creative self-efficacy (*β* = 0.42, *p* < 0.001) and creative deviance (*β* = 0.64, *p* < 0.001), and that creative self-efficacy partially mediated that relationship. Transformational leadership strengthens the effect of narcissism on creative self-efficacy, and there is a positive indirect relationship between employee narcissism and creative deviance through creative self-efficacy when transformational leadership is high. These findings extend the understanding of the antecedents of creative deviance by showing the relations between employee narcissism and creative deviance. The study also contributes to the literature of mediating role of creative self-efficacy and the moderating role of transformational leadership to explain the relationship between employee narcissism and creative deviance.

## Introduction

Narcissism is characterized as a “complex of personality traits that involve grandiosity, superiority, dominance, self-focus, focus on success and the demand to be admired” (Judge et al., 2006; Spurk et al., 2016). Generally, narcissistic people are considered destructive, abusive, or toxic due to their significant harm to or exploitation of others. Employee narcissism has raised concerns in management practice and theoretical research. For instance, employee narcissism is positively related to abusive leadership (Rosenthal and Pittinsky, 2006) and counterwork behavior (CWB; Penney and Spector, 2002; Judge et al., 2006). While employee narcissism is generally considered socially undesirable, the recent dark personality literature has called for studying narcissism from a more complex, multidimensional perspective (Fox and Freeman, 2011; Jonason et al., 2014). There is a small but growing body of literature suggesting that narcissism can also have a potential benefit for organizations by way of some constructive aspects (such as innovation behavior, prosocial behavior, and whistle blowing; Resick et al., 2009; Gerstner et al., 2013; Konrath et al., 2016; Zhang et al., 2017; Jalan, 2020).

Creative deviance occurs when employees violate their managers’ orders to stop working on a new idea (Mainemelis, 2010). Previous studies suggest that narcissistic people have a self-enhanced motivation to demonstrate creative thinking (Smith and Webster, 2018). Narcissists are also considered exempt from organizational, cultural, and interpersonal rules and norms and they also exhibit deviant behaviors (Judge et al., 2006; Adams et al., 2014; Shah et al., 2020). Thus, will narcissistic employees defy a leader’s will to implement creative deviance? Additionally, what is the internal mechanism underlying this relation? Although there is some evidence that emphasizes the positive association between a narcissistic personality and innovative behavior (Resick et al., 2009; Gerstner et al., 2013; Smith and Webster, 2018), the question of whether narcissists are creative remains open, as some research shows that narcissists do not truly generate more creative ideas (Goncalo et al., 2010). Generally, narcissistic individuals always feel entitled and believe that they are extremely gifted. Therefore, the sense of entitlement that accompanies narcissism will likely lead them to engage in creative deviance to prove their superiority. To date, the relationship between employee narcissism and creative deviance has attracted little attention in existing research. To answer these questions and fill this research gap, we draw on narcissism theory and hypothesize that narcissistic employees will believe in their ability to produce creative outcomes (creative self-efficacy, CSE), which promotes creative deviance.

**Figure 1:**
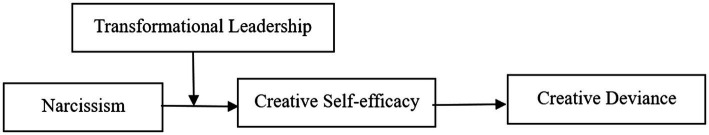
Theoretical model.

Trait activation theory has shown that leadership is a vital situational cue that can activate followers’ trait expressions (Tett and Burnett, 2003; Colbert and Witt, 2009; Greenbaum et al., 2014; Song and Lee, 2020). Transformational leaders encourage follower confidence to perform beyond expectations and facilitate new idea generation among followers (Dvir et al., 2002; Shin and Zhou, 2003). Hence, narcissistic followers appear to feel good about expressing creativity-related traits (e.g., self-expression and self-enhancement) under transformational leadership. In this paper, we argue that transformational leadership strengthens narcissists’ creative self-efficacy and further triggers creative deviance. Finally, we develop a theoretical framework in which CSE serves as a mediator of the effect of employee narcissism and creative deviance, and transformational leadership serves as a moderator of this effect. Our conceptual model is depicted in Figure 1.

The current study makes three primary contributions to the extant literatures. First, most previous studies on narcissism primarily focused on narcissistic toxicity and its dark side (Judge et al., 2006; Lau and Marsee, 2013; Lobbestael et al., 2014; Grijalva and Newman, 2015; Shah et al., 2020), whereas this study emphasizes the “nonnegative consequence” of narcissism by investigating the positive relationship between employee narcissism and creative deviance. Second, previous studies have shown that specific self-efficacy mediates the relationship between narcissism and behaviors (Al-Ghazali and Afsar, 2020). To our knowledge, no studies to date have investigated the mediating effect of CSE on the relationship between employee narcissism and creative deviance. By examining CSE as a mediator, our work expands the theoretical understanding of the creative deviance literature by identifying and testing a trait activation mechanism that links narcissism to creative deviance. Third, by echoing the call for a more complete view of the contextual influences in the research on narcissism (Nevicka et al., 2018; Mao et al., 2021), we explore in our theoretical framework the moderating effect of transformational leadership, and we expand knowledge in the creativity literature to understand how creative deviance can be affected by the joint influence of individual and leader factors.

## Theoretical framework and research hypotheses

### Narcissism and creative deviance

We suggest that narcissism is positively related to creative deviance. Creative deviance involves two critical components: a creative dimension and a deviance dimension (Cohen, 1965; Robinson and Bennett, 1995). First, because narcissism is associated with a need for self-expression and self-enhancement, narcissists are likely to produce creative thoughts and participate in creative activities (Wallace and Baumeister, 2002; Martinsen et al., 2019). In previous studies, a robust body of literature has demonstrated a positive link between narcissism and creativity (Wallace and Baumeister, 2002; Martinsen et al., 2019). Second, narcissists have a sense of entitlement, and they may believe that engaging in deviant behavior is acceptable (Grijalva and Newman, 2015). Therefore, narcissists consider themselves exempt from organizational, cultural, and interpersonal rules and norms and then implement deviant behaviors (Judge et al., 2006; Adams et al., 2014; Shah et al., 2020). Furthermore, narcissistic individuals have grandiose self-views and tend to overestimate their abilities and positive outcomes of their behaviors, resulting in overconfidence and risk-taking (Campbell et al., 2004; Buyl et al., 2019). Tenzer and Yang (2019) suggest that risk propensity promotes creative deviance by generating radical new ideas and overestimating the likelihood of success in risky endeavors. As such, narcissism as an antecedent to creative deviance is not surprising. Similarly, Shukla and Kark (2020) suggest that personality traits such as narcissism can lead to creative deviance. Therefore, this study hypothesizes the following:

*Hypothesis 1*: Narcissism is positively related to creative deviance.

### Narcissism and creative self-efficacy

Narcissists have a strong sense of entitlement and a constant need for attention and admiration (Bogart et al., 2004), and they tend to feel optimistic and confident in their accomplishments (Bogart et al., 2004). Although few studies have focused on the relationship between narcissism and creative self-efficacy, previous studies have shown that narcissistic employees display high levels of occupational self-efficacy and entrepreneurial self-efficacy (Hirschi and Jaensch, 2015; Credo et al., 2016). Goncalo et al. (2010) suggest that narcissists are more likely to perceive themselves as being more creative than others. Hence, it can be inferred that narcissists will have more confidence when handling problems requiring creative thinking. Based on the theoretical analysis above, we hypothesize the following:

*Hypothesis 2*: Narcissism is positively related to creative self-efficacy.

### Creative self-efficacy and creative deviance

We propose that employees with creative self-efficacy are more likely to engage in creative deviance. First, CSE can promote individual creativity. Creative activities need perseverance in the face of challenges, and creativity combined with strong efficacy beliefs can enhance an individual’s perseverance to facilitate creative processes (Tierney and Farmer, 2002). Hence, CSE can motivate individuals to overcome obstacles through trial-and-error experimentation in creative activities (Tierney and Farmer, 2011). A considerable number of studies have documented creative self-efficacy as a critical ascendant of creativity (Tierney and Farmer, 2002, 2011; Gong et al., 2009; Wang et al., 2014, 2018). Furthermore, CSE can also lead to deviant behavior. CSE makes people believe that they are capable of setting a challenging goal beyond basic requirements and exert more effort in pursuit of these goals. Many studies suggest that self-efficacy is a main contributor to workplace deviance (Redmond et al., 1993; Morrison and Phelps, 1999; Vadera et al., 2013). Thus, individuals with CSE are more likely to engage in creative deviance because of their beliefs that they can produce creative outcomes. Thus, we hypothesized the following:

*Hypothesis 3*: Creative self-efficacy is positively related to creative deviance.

### Mediating effects of creative self-efficacy

Generally, narcissists are driven by pro-self-motivations such as self-worth and self-enhancement, which may maximize their superiority relative to others (Elliot and Thrash, 2001; Zeigler-Hill and Besser, 2013; Grijalva and Zhang, 2016; Shukla and Kark, 2020). People with pro-self-motivation have been noted to enact creative deviance (Shukla and Kark, 2020). Thus, integrating narcissism theory (Emmons, 1987) and trait activation theory (Tett and Burnett, 2003), pro-self-motivated narcissists may feel that “they can” generate creative ideas (display high levels of creative self-efficacy), and this may further inspire narcissists to engage in creative deviance (Lin and Chen, 2012). In previous studies, creative self-efficacy is a malleable mindset and can be seen as an important mediator between individual dispositions and creative performance (Gong et al., 2009). Thus, we hypothesize that:

*Hypothesis 4*: Creative self-efficacy mediates the relationship between narcissism and creative deviance.

### Moderating effects of transformational leadership

Transformational leadership was conceptualized as influencing followers by expanding and elevating their goals and providing them with the confidence to perform beyond predetermined expectations (Dvir et al., 2002). Avolio et al. (1999) suggest that transformational leaders can inspire their subordinates by creating an idealized vison, challenging the status quo, engaging in intellectual stimulation, and focusing on their development (Bass, 1985; Bass and Avolio, 1994). Transformational leaders provide followers with job autonomy to decide how and when to perform their tasks and support innovation (Avolio and Bass, 1995; Jung, 2001; Breevaart et al., 2014; Gilbert et al., 2017). Employees with job autonomy have more practical opportunities to engage in creative deviance (Mainemelis, 2010). Previous studies suggest that followers under transformational leadership are more likely to behave in a way that changes the status quo and violate work and organizational norms (e.g., creativity, constructive deviance, extrarole behaviors, voice, prosocial rule breaking, job crafting; Shin and Zhou, 2003; Detert and Burris, 2007; Gumusluoglu and Ilsev, 2009; Vadera et al., 2013; Jaiswal and Dhar, 2015; Mittal and Dhar, 2015; Qu et al., 2015; Hetland et al., 2018). Based on trait activation theory, an individual’s personality traits will be activated by situational cues and subsequently manifest themselves behaviorally (Tett and Burnett, 2003; Tett et al., 2021). Regarding the narcissistic personality, narcissists have a great sense of self-expression and self-enhancement, which help them claim uniqueness. As a result, narcissists enjoy engaging in creativity activities (Wallace and Baumeister, 2002; Martinsen et al., 2019). Transformational leadership can be regarded as a trait-relevant cue to trigger the free expression of creative activities for narcissists. Transformational leaders appear to motivate their narcissist followers’ self-expression to think in novel ways despite intellectual stimulation, and transformational leaders may also satisfy narcissist followers’ personal development through individualized consideration. Thus, faced with transformational leadership, narcissists tend to be aware of potential rewards and more confident in engaging in creativity. In contrast, narcissistic subordinates who work under close monitoring, such as authoritarian and abusive narcissistic subordinates, may feel unsafe to bring up new ideas that challenge the authority of the leader (Liu et al., 2016; Zheng et al., 2020).

Moreover, we expect that transformational leadership will moderate the indirect effects of narcissism on creative deviance through CSE. Leadership is a vital situational cue that can activate trait expression (Colbert and Witt, 2009; Greenbaum et al., 2014; Song and Lee, 2020). Transformational leaders create a strong contextual situation that motivates followers to go beyond by doing more than they feel is possible (Mainemelis, 2010). Thus, when a narcissist’s immediate supervisor is high in transformational leadership, narcissistic followers are likely intrinsically satisfied and feel more self-efficacious in engaging in creative deviance against the leader’s order.

In summary, we have developed a theoretical foundation for the mediating role of creative self-efficacy and the moderating role of transformational leadership. That is, since creative self-efficacy mediates the relationship between narcissism and creative deviance, transformational leadership moderates the relationship between narcissism and creative self-efficacy. We thus propose that the indirect effect that narcissism has on creative deviance *via* creative deviance depends on transformational leadership.

*Hypothesis 5*: Transformational leadership moderates the effect of narcissism on CSE such that the relationship is stronger (versus weaker) among employees under higher (versus lower) transformational leadership.

*Hypothesis 6*: Transformational leadership moderates the indirect effects of narcissism on creative deviance such that the relationship is stronger (versus weaker) among employees under higher (versus lower) transformational leadership.

## Materials and methods

### Samples and procedure

Participants were recruited for a three-wave field study from 6 different corporations in China. With ethical approval from IRB of Changchun Guanghua University, we selected the companies to be surveyed based on MBA schools and obtained the survey sample through a simple random sampling method by the HR managers of the companies. One of the authors contacted the human resources director of the companies to introduce the research project to inform the purpose of our study. Participants were informed of the purpose of the study and responded to relevant questions on a voluntary basis. The Time 1 survey measured gender, age, education, tenure, and narcissism. 704 participants with 590 fully completed questionnaires. 474 employees completed the Time 2 survey, wherein they provided their CSE and their immediate leader’s transformational leadership (response rate of 80.33%). At Time 3, leaders rated their followers’ creative deviance of their subordinates, and we received 107 responses (response rate of 93.84%). Finally, 446 valid survey questionnaires were obtained. Participants were compensated for their time.

Of this sample, 232 participants (52%) were female, 214 participants (48%) were male, 171 participants (38.3%) were 21–25 years old, 139 participants (31.1%) were 26–30 years old, a total of 63.3% had a college degree or higher, and approximately 69.5% of the participants had 1–5 years of work experience.

### Measures

We followed the suggestions recommended by Brislin (1980) to ensure that all measures were accurately translated from English to Chinese. Additionally, the narcissism measurement was coded 1 for narcissism-consistent responses and 0 for narcissism-inconsistent responses. Creative self-efficacy, creative deviance, and transformational leadership were measured using a 5-point Likert scale (1 = strongly disagree, 5 = strongly agree).

#### Narcissism

Narcissism was measured with the 16-item version of the Narcissistic Personality Inventory (NPI) scale (Ames et al., 2006). The following is an example item: “I show others how special I am” (Cronbach’s alpha = 0.85).

#### Creative self-efficacy

CSE was measured with a 3-item scale from Tierney and Farmer (2002). The following is an example item: “I have confidence in my ability to solve problems creatively” (Cronbach’s alpha = 0.82).

#### Creative deviance

Creative deviance was measured with a 9-item scale from Lin et al. (2016). The following is an example item: “I continued to improve some of the new ideas, although they did not receive my supervisor’s approval.” (Cronbach’s alpha = 0.86).

#### Transformational leadership

Transformational leadership was measured with a 16-item scale from (Avolio et al., 1999). The following is an example item: “My leader emphasizes the importance of vision.” (Cronbach’s alpha = 0.88).

#### Control variables

Based on suggestions of control variables (Carlson and Wu, 2012; Bernerth and Aguinis, 2016), gender, age, education, and tenure were controlled since they are related to creative deviance (Lin et al., 2016; Liu and Zhou, 2020).

## Results

### Confirmatory factor analysis

A confirmatory factor analysis (CFA) was conducted and is presented in Table 1. According to the criteria in existing studies of organizational behavior (CMIN/DF < 3, RMESA<0.06, GFI > 0.90, IFI > 0.90, TLI > 0.90), the four-factor model (narcissism, creative self-efficacy, creative deviance, and transformational leadership) provided a significantly better fit than any other model: chi-square (*χ2*) = 445.25, degrees of freedom *(df*) = 230, CFI = 0.91, IFI =0.91, TLI = 0.90, and RMSEA = 0.05. We examined the average variance extracted (AVE) and composite reliability (CR) to further evaluate the convergent validity. According to criteria considered acceptable in previous studies (Bagozzi and Yi, 1988). The convergent validity was at an acceptable level as shown in Table 1. Hair (2009) suggest that discriminant validity is accepted when the maximum shared variance (MSV) is smaller than AVE. As shown in Table 2, the discriminant validity value was acceptable.

**Table 1:** Confirmatory factor analysis.

Measurement Models	**χ** ^2^	*df*	CFI	TLI	IFI	RMSEA
Four-factor	445.25	230	0.91	0.91	0.90	0.051
Three-factor (combining transformational leadership and creative deviance into one factor)	656.87	232	0.84	0.84	0.83	0.077
Two-factor (combining transformational leadership, creative self-efficacy, and creative deviance into one factor)	803.63	245	0.81	0.79	0.81	0.073
One-factor (combining all items into one factor)	937.64	248	0.62	0.62	0.60	0.100

CFI = comparative fit index; IFI = incremental fit index; TLI = Tucker-Lewis index; RMSEA = root mean square error of approximation.

**Table 2:** Results of the descriptive statistical analysis.

Variables	M	SD	CR	AVE	MSV	1	2	3	4	5	6	7	8
1. Gender	0.48	0.50											
2. Age	3.10	1.16				−0.08							
3. Education	2.73	0.79				0.01	0.03						
4. Tenure	2.67	1.30				0.04	−0.03	0.62^**^					
5. Narcissism (Time 1)	0.59	0.30	0.78	0.64	0.28	−0.06	0.00	0.06	0.07	0.85			
6. Creative Self-efficacy (Time 2)	3.84	0.65	0.88	0.70	0.28	−0.11^*^	0.10^*^	−0.064	0.01	0.42^**^	0.82		
7. Creative Deviance (Time 3)	3.57	1.01	0.91	0.52	0.01	−0.11^*^	0.02	0.003	0.10^*^	0.52^**^	0.55^**^	0.86	
8. Transformational Leadership (Time 2)	2.78	1.05	0.92	0.58	0.01	−0.03	0.02	0.03	0.09	0.10^*^	0.01	0.12^*^	0.88

*n = *446; Cronbach’s alpha reliabilities are displayed on the diagonal; *p < 0.05, **p < 0.01.

### Common method bias testing

CMB (CMB) was assessed by Harman’s single-factor test (Podsakoff et al., 2003) were used to examine common method bias (CMB). Result show that the total variance of a single component of four theoretical constructs was 23.31%, much below the recommended minimum of 50%. Therefore, CMB is not a serious problem.

### Descriptive statistics

The means, standard deviations, and Pearson’s correlation analysis are indicated in Table 2. In line with the prediction, the results demonstrated a positive relationship between narcissism (Time 1) and creative self-efficacy (Time 2; *r* = 0.42, *p* < 0.01) and creative deviance (Time 3; *r* = 0.52, *p* < 0.01). In addition, creative self-efficacy (Time 2) was positively correlated with creative deviance (Time 3; *r* = 0.55, *p* < 0.01). Transformational leadership (Time 2) was positively correlated with creative deviance (Time 3; *r* = 0.12, *p* < 0.05). We also performed a normality distribution test. Most of the question items had skewness coefficients less than 3 in absolute value and kurtosis coefficients much less than 10 in absolute value, indicating that the sample data conformed to a multivariate normal distribution (Kline, 1998).

### Hypothesis testing

First, we examined the mediation of creative self-efficacy. The analysis results are reported in Table 3: narcissism positively and significantly predicted creative deviance (*β* = 0.64, *p* < 0.001) and creative self-efficacy (*β* = 0.42, *p* < 0.001), and creative self-efficacy was positively associated with creative deviance (*β* = 0.40, *p* < 0.001), supporting Hypotheses 1–3. Multiple regression analysis shows that VIF < 3, indicating that the regression model does not have severe multicollinearity. A bias-corrected bootstrapping technique [*n* = 5,000; PROCESS, Model 4; Hayes (2013)] was further used to test the mediation effect. Supporting Hypothesis 4, we found a significant indirect relationship of narcissism with creative deviance (indirect effect = 0.21, *SE* = 0.04, 95% CI = [0.13, 0.30]).

**Table 3:** Moderated mediation analysis.

Variables	Creative self-efficacy			Creative deviance		
	Model 1	Model 2	Model 3	Model 4	Model 5	Model 6
Intercept	3.26^***^	3.32^***^	3.76^***^	11.25^***^	5.64^***^	3.55^***^
Age	−0.14	−0.14	−0.14	−0.06	−0.07	−0.01
Gender	−0.07	−0.07	−0.07	−0.11	−0.07	−0.04
Education	0.10^*^	0.10^*^	0.10^*^	0.02	0.02	−0.01
Tenure	0.07	0.08	0.08	0.14^*^	0.11^*^	0.08
Narcissism	0.42^***^	0.42^***^	0.08		0.64^***^	0.34^***^
Creative self-efficacy						0.40^***^
Transformational leadership		−0.04	−0.29^*^			
Narcissim × Transformational leadership			0.46^**^			
*R* ^2^	0.20	0.21	0.22	0.02	0.29	0.41
Adjusted *R*^2^	0.19	0.19	0.21	0.01	0.28	0.40
F	22.43^***^	18.82^***^	17.67^***^	2.96^*^	34.82^***^	50.57^***^

*n* = 446; ^*^p < 0.05, ^**^p < 0.01, ^***^p < 0.001.

**Figure 2:**
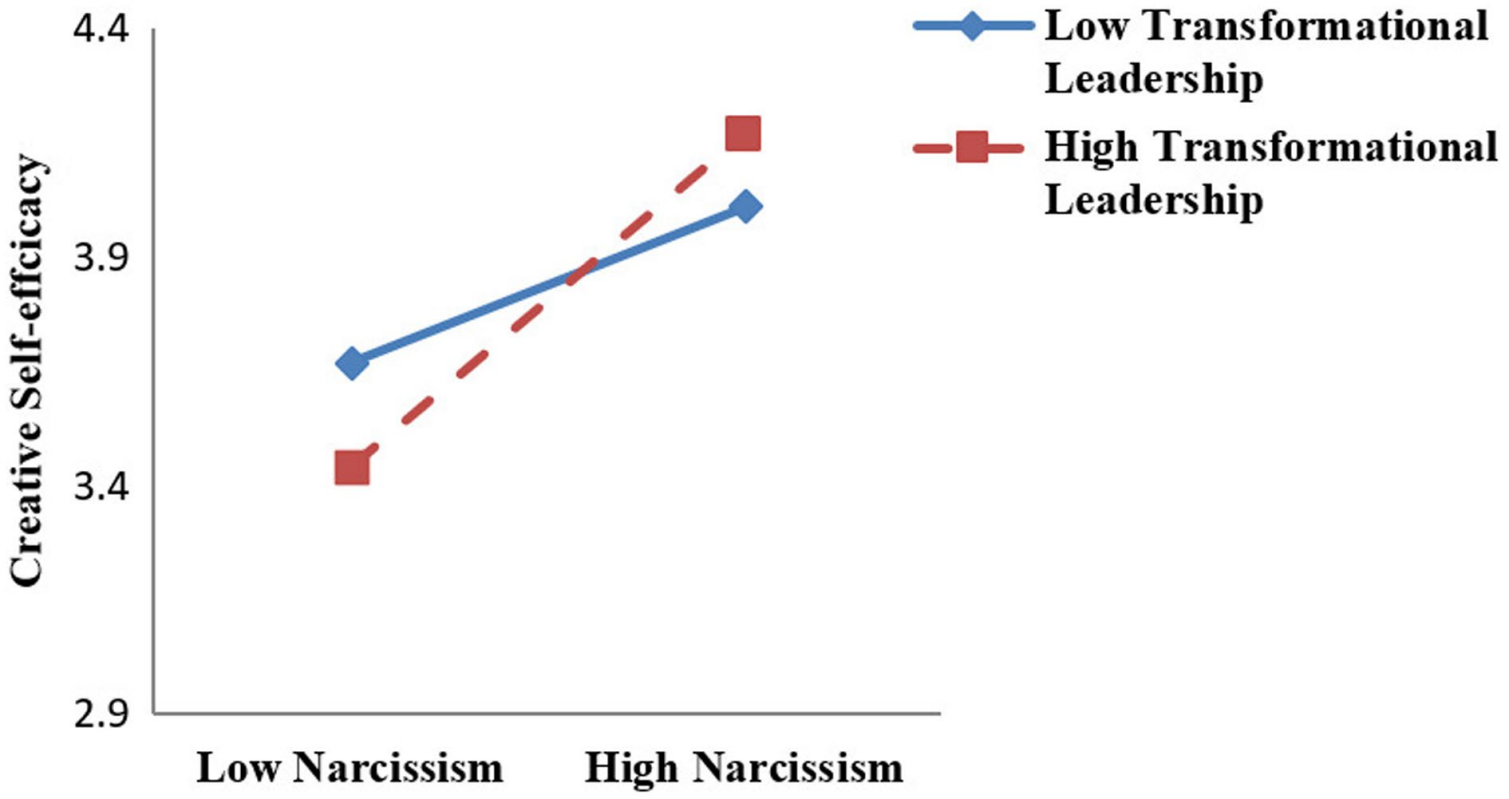
Moderating effect of transformational leadership on the relationship between narcissism and creative self-efficacy.

Second, we tested the moderated model relying on PROCESS syntax (model 7) to examine the moderating role of transformational leadership in the positive relationship between narcissism and creative self-efficacy (Edwards and Lambert, 2007; Hayes, 2013). As illustrated in model 3 in Table 3, the interaction term between narcissism and transformational leadership was significant (*β* = 0.46, *p* < 0.01). We then tested the simple slope tests and plotted the interactive effects in Figure 2. Simple slopes analysis indicates that the effect of narcissism was stronger when transformational leadership was high (*β* = 0.07, *SE* = 0.08, *p* < 0.001) than when it was low (*β* = 0.03, *SE* = 0.08, *p* < 0.001; see Figure 2). Thus, Hypothesis 5 is supported.

Furthermore, we test the conditional indirect effect of transformational leadership on the relationship between narcissism and creative deviance. As illustrated in Table 4, the conditional indirect effect was both positive and statistically significant for a high level of transformational leadership (+1 *SD*, indirect effect = 0.27, *SE* = 0.06, 95% CI = [0.164, 0.406]) and a low level of transformational leadership (−1 *SD*, indirect effect = 0.14, *SE* = 0.04, 95% CI = [0.065, 0.247]). and the difference between the two indirect effects was also significant (*∆β* = 0.13, *SE* = 0.06, 95% CI = [0.011, 0.258]). Thus, Hypothesis 6 is supported.

**Table 4:** Conditional indirect effects.

Moderator	Level	Effect	*Boot SE*	*Boot p*	CI
Transformational leadership	Low (−1 SD)	0.039	0.084	0.000	[0.023, 0.056]
	High (+1 SD)	0.074	0.082	0.000	[0.058, 0.090]

*n = *446; CI = confidence interval. Bootstrapping repetitions *N* = 5,000.

## Discussion

### Theoretical implications

Creative deviance has gradually attracted widespread attention from researchers (Lin et al., 2016; Tenzer and Yang, 2019; Liu and Zhou, 2020; Shukla and Kark, 2020; Liu et al., 2022). However, many important questions remain. Drawing on narcissism theory (Emmons, 1987) and trait activation theory (Tett and Burnett, 2003), our results explain how narcissism promotes creative deviance *via* a trait-activation mechanism (self-efficacy). Furthermore, immediate supervisors’ transformational leadership moderates the relation between narcissism and the indirect effect between narcissism and creative deviance. The current set of studies makes follow contributions.

First, by echoing calls for exploring when and how personality traits influence creative deviance (Mainemelis, 2010), this study finds that narcissism has a significantly positive effect on creative deviance. These results contribute to the literature on creativity and suggest that individuals with personality traits such as narcissism may be more likely to violate managerial orders to engage in creative activities. Because narcissists tend to be risk seeking and have a need for self-expression and self-enhancement, which impels creative deviance. Although previous studies suggest that narcissists are creative (Smith and Webster, 2018), this result is consistent with these findings and extends them, finding that narcissists are more willing to engage in creativity that violates a leader’s order.

Second, the findings support CSE serving as a mediator of the narcissism-creative deviance relationship. This result shows that narcissism will increase CSE and thereby foster creative deviance. In line with narcissism theory (Emmons, 1987) and trait activation theory (Emmons, 1987; Gist and Mitchell, 1992; Tett and Burnett, 2003), we can further explain this result in that narcissists may be triggered by self-expression and self-enhancement and display high levels of creative self-efficacy, which further inspires narcissists to engage in creative deviance. The present work seems to extend the work of Martinsen et al. (2019) by unlocking the mediation mechanism between narcissism and creative deviance. This result will deepen our understanding of narcissism by showing that narcissists will promote self-belief and confidence in performing risky innovation activities.

Third, our findings show that immediate supervisors’ transformational leadership as a boundary condition strengthens in direct relation and indirect relation between narcissism and creative deviance. Regarding trait activation theory, our study supports the idea that transformational leadership may activate the free expression of creativity-related traits of narcissists, which in turn promote narcissists’ engagement in creative deviance. Although previous research has shed some light on the positive link between narcissism and creativity, few studies have empirically investigated the boundary condition for the relationship between narcissism and creativity. This result answers the call for more empirical investigations of moderators of the relationships between narcissism and organizational outcomes (LeBreton et al., 2018). Furthermore, previous studies have mainly focused on situational factors that stimulate the dark side of narcissism (Liu et al., 2017; Shah et al., 2020). Considering the potential constructive facets of creative deviance for organizations (Mainemelis, 2010; Shukla and Kark, 2020), the current findings seem to go beyond these studies by showing that transformational leadership can turn toxic narcissists into “good soldiers” and trigger narcissists’ creative deviance.

### Managerial implications

The findings of this study highlight several significant managerial implications for organizations. First, the findings suggest that narcissistic traits increase the likelihood of engagement in creative deviance. Because creative deviance is constructive and beneficial for organizations, organizations should evaluate narcissistic employees more comprehensively in recruiting and performance evaluations. Second, CSE mediates the relationship between narcissism and creative deviance. Narcissistic employees tend to be self-motivated to perform risky creativity. Organizations should try to involve those employees and provide a more inclusive culture for them. Third, our study found that transformational leadership is a boundary condition that triggers narcissists’ creative deviance. Thus, organizations should select leaders based on their potential as transformational leaders and provide training programs to supervisors to help them develop transformational leadership.

### Limitations and future research

First, we incorporate CSE as a mediator to explain the relationship between narcissism and creative deviance, but many other variables and theories could explain this process. Therefore, future research is necessary to identify other mechanisms underlying the relationship between narcissism and creative deviance. Second, our data were collected in China only, and we did not consider cultural aspects in our model. Future research could also explore the generalizability of our results to other cultures and countries.

## Data availability statement

The datasets presented in this article are not readily available due to participants’ privacy. Requests to access the datasets should be directed to ZC, cui_zilong@hotmail.com.

## Ethics statement

The studies involving human participants were reviewed and approved by Jilin University of Finance and Economics and Changchun Guanghua University. The patients/participants provided their written informed consent to participate in this study.

## Author contributions

ZC contributed to the study’s theoretical foundation, model development, and research design. KZ contributed to the literature research. ZC and KZ contributed to the analysis, interpretation of the data, and drafting of the manuscript. All authors contributed to the article and approved the submitted version.

## Funding

This study was supported by the Doctoral Research Initiation Project of Jilin University of Finance and Economics (RES0005525).

## Conflict of interest

The authors declare that the research was conducted in the absence of any commercial or financial relationships that could be construed as a potential conflict of interest.

## Publisher’s note

All claims expressed in this article are solely those of the authors and do not necessarily represent those of their affiliated organizations, or those of the publisher, the editors and the reviewers. Any product that may be evaluated in this article, or claim that may be made by its manufacturer, is not guaranteed or endorsed by the publisher.

## References

[ref1] Adams J. M. Florell D. Burton K. A. Hart W. (2014). Why do narcissists disregard social-etiquette norms? A test of two explanations for why narcissism relates to offensive-language use. Pers. Individ. Differ. 58, 26–30. doi: 10.1016/j.paid.2013.09.027

[ref2] Al-Ghazali B. M. Afsar B. (2020). Narcissism and entrepreneurial intentions: the roles of entrepreneurial self-efficacy and environmental complexity. J. High Technol. Manag. Res. 32:100395. doi: 10.1016/j.hitech.2020.100395

[ref3] Ames D. R. Rose P. Anderson C. P. (2006). The NPI-16 as a short measure of narcissism. J. Res. Pers. 40, 440–450. doi: 10.1016/j.jrp.2005.03.002

[ref4] Avolio B. J. Bass B. M. (1995). Individual consideration viewed at multiple levels of analysis: a multi-level framework for examining the diffusion of transformational leadership. Leadersh. Q. 6, 199–218. doi: 10.1016/1048-9843(95)90035-7

[ref5] Avolio B. J. Bass B. M. Jung D. I. (1999). Re-examining the components of transformational and transactional leadership using the multifactor leadership. J. Occup. Organ. Psychol. 72, 441–462. doi: 10.1348/096317999166789

[ref6] Bagozzi R. P. Yi Y. (1988). On the evaluation of structural equation models. J. Acad. Mark. Sci. 16, 74–94. doi: 10.1007/BF02723327

[ref7] Bass B. M. (1985). Leadership: good, better, best. Organ. Dyn. 13, 26–40. doi: 10.1016/0090-2616(85)90028-2

[ref8] Bass B. M. Avolio B. J. (1994). Transformational leadership and organizational culture. Int. J. Public Adm. 17, 541–554. doi: 10.1080/01900699408524907

[ref9] Bernerth J. B. Aguinis H. (2016). A critical review and best-practice recommendations for control variable usage. Pers. Psychol. 69, 229–283. doi: 10.1111/peps.12103

[ref10] Bogart L. M. Benotsch E. G. Pavlovic J. D. P. (2004). Feeling superior but threatened: the relation of narcissism to social comparison. Basic Appl. Soc. Psychol. 26, 35–44. doi: 10.1207/s15324834basp2601_4

[ref11] Breevaart K. Bakker A. Hetland J. Demerouti E. Olsen O. K. Espevik R. (2014). Daily transactional and transformational leadership and daily employee engagement. J. Occup. Organ. Psychol. 87, 138–157. doi: 10.1111/joop.12041

[ref12] Brislin R. W. (1980). “Cross-cultural research methods,” in Environment and culture. eds. Altman I. Rapoport A. Wohlwill J. F. (Boston, MA: Springer), 47–82.

[ref13] Buyl T. Boone C. Wade J. B. (2019). CEO narcissism, risk-taking, and resilience: an empirical analysis in U.S. commercial banks. J. Manag. 45, 1372–1400. doi: 10.1177/0149206317699521

[ref14] Campbell W. K. Goodie A. S. Foster J. D. (2004). Narcissism, confidence, and risk attitude. J. Behav. Decis. Mak. 17, 297–311. doi: 10.1002/bdm.475

[ref15] Carlson K. D. Wu J. (2012). The illusion of statistical control: control variable practice in management research. Organ. Res. Methods 15, 413–435. doi: 10.1177/1094428111428817

[ref16] Cohen A. K. (1965). The sociology of the deviant act: anomie theory and beyond. Am. Sociol. Rev. 30, 5–14. doi: 10.2307/2091770 14247328

[ref17] Colbert A. E. Witt L. A. (2009). The role of goal-focused leadership in enabling the expression of conscientiousness. J. Appl. Psychol. 94, 790–796. doi: 10.1037/a0014187, PMID: 19450014

[ref18] Credo K. R. Lanier P. A. Matherne C. F. Cox S. S. (2016). Narcissism and entitlement in millennials: the mediating influence of community service self efficacy on engagement. Pers. Individ. Differ. 101, 192–195. doi: 10.1016/j.paid.2016.05.370

[ref19] Detert J. R. Burris E. R. (2007). Leadership behavior and employee voice: is the door really open? Acad. Manag. J. 50, 869–884. doi: 10.5465/amj.2007.26279183

[ref20] Dvir T. Eden D. Avolio B. J. Shamir B. (2002). Impact of transformational leadership on follower development and performance: a field experiment. Acad. Manag. J. 45, 735–744. doi: 10.5465/3069307

[ref21] Edwards J. R. Lambert L. S. (2007). Methods for integrating moderation and mediation: a general analytical framework using moderated path analysis. Psychol. Methods 12, 1–22. doi: 10.1037/1082-989X.12.1.1, PMID: 17402809

[ref22] Elliot A. J. Thrash T. M. (2001). Achievement goals and the hierarchical model of achievement motivation. Educational Psychology Review 13, 139–156. doi: 10.1023/A:1009057102306

[ref23] Emmons R. A. (1987). Narcissism: theory and measurement. J. Pers. Soc. Psychol. 52, 11–17. doi: 10.1037/0022-3514.52.1.11, PMID: 3820065

[ref24] Fox S. Freeman A. (2011). “Narcissism and the deviant citizen: a common thread in CWB and OCB,” in The role of individual differences in occupational stress and well being. eds. Pamela L. P. Daniel C. G. (Bingley, UK: Emerald Group Publishing Limited), 151–196.

[ref25] Gerstner W. C. König A. Enders A. Hambrick D. C. (2013). CEO narcissism, audience engagement, and organizational adoption of technological discontinuities. Adm. Sci. Q. 58, 257–291. doi: 10.1177/0001839213488773

[ref26] Gilbert M. H. Dagenais-Desmarais V. St-Hilaire F. (2017). Transformational leadership and autonomy support management behaviors. Leadersh. Organ. Dev. J. 38, 320–332. doi: 10.1108/LODJ-08-2015-0173

[ref27] Gist M. E. Mitchell T. R. (1992). Self-efficacy: a theoretical analysis of its determinants and malleability. Acad. Manag. Rev. 17, 183–211. doi: 10.5465/amr.1992.4279530

[ref28] Goncalo J. A. Flynn F. J. Kim S. H. (2010). Are two narcissists better than one? The link between narcissism, perceived creativity, and creative performance. Pers. Soc. Sci. Bull. 36, 1484–1495. doi: 10.1177/0146167210385109, PMID: 20947771

[ref29] Gong Y. Huang J. C. Farh J. L. (2009). Employee learning orientation, transformational leadership, and employee creativity: the mediating role of employee creative self-efficacy. Dev. Learn. Organ. 52, 765–778. doi: 10.5465/amj.2009.43670890

[ref30] Greenbaum R. L. Hill A. Mawritz M. B. Quade M. J. (2014). Employee machiavellianism to unethical behavior: the role of abusive supervision as a trait activator. J. Manag. 43, 585–609. doi: 10.1177/0149206314535434

[ref31] Grijalva E. Newman D. A. (2015). Narcissism and counterproductive work behavior (CWB): meta-analysis and consideration of collectivist culture, big five personality, and narcissism's facet structure. Appl. Psychol. 64, 93–126. doi: 10.1111/apps.12025

[ref32] Grijalva E. Zhang L. (2016). Narcissism and self-insight: a review and meta-analysis of narcissists’ self-enhancement tendencies. Personal. Soc. Psychol. Bull. 42, 3–24. doi: 10.1177/0146167215611636, PMID: 26542339

[ref33] Gumusluoglu L. Ilsev A. (2009). Transformational leadership, creativity, and organizational innovation. J. Bus. Res. 62, 461–473. doi: 10.1016/j.jbusres.2007.07.032

[ref34] Hair J. F. (2009). Multivariate data analysis: A global perspective. Upper Saddle River, NJ: Prentice Hall.

[ref35] Hayes A. F. (2013). Introduction to mediation, moderation, and conditional process analysis: A regression-based approach. New York, NY: Guilford Press.

[ref36] Hetland J. Hetland H. Bakker A. B. Demerouti E. (2018). Daily transformational leadership and employee job crafting: the role of promotion focus. Eur. Manag. J. 36, 746–756. doi: 10.1016/j.emj.2018.01.002

[ref37] Hirschi A. Jaensch V. K. (2015). Narcissism and career success: occupational self-efficacy and career engagement as mediators. Pers. Individ. Differ. 77, 205–208. doi: 10.1016/j.paid.2015.01.002

[ref38] Jaiswal N. K. Dhar R. L. (2015). Transformational leadership, innovation climate, creative self-efficacy and employee creativity: a multilevel study. Int. J. Hosp. Manag. 51, 30–41. doi: 10.1016/j.ijhm.2015.07.002

[ref39] Jalan I. (2020). Treason or reason? Psychoanalytical insights on whistleblowing. Int. J. Manag. Rev. 22, 249–263. doi: 10.1111/ijmr.12224

[ref40] Jonason P. K. Wee S. Li N. P. (2014). Thinking bigger and better about bad apples: evolutionary industrial/organizational psychology and the dark triad. Ind. Organ. Psychol. 7, 117–121. doi: 10.1111/iops.12118

[ref41] Judge T. A. LePine J. A. Rich B. L. (2006). Loving yourself abundantly: relationship of the narcissistic personality to self- and other perceptions of workplace deviance, leadership, and task and contextual performance. J. Appl. Psychol. 91, 762–776. doi: 10.1037/0021-9010.91.4.762, PMID: 16834504

[ref42] Jung D. I. (2001). Transformational and transactional leadership and their effects on creativity in groups. Creat. Res. J. 13, 185–195. doi: 10.1207/S15326934CRJ1302_6

[ref02] Kline R. B. (1998). Principles and practice of structural equation modeling. New York, NY: Guilford Press.

[ref43] Konrath S. Ho M. H. Zarins S. (2016). The strategic helper: narcissism and prosocial motives and behaviors. Curr. Psychol. 35, 182–194. doi: 10.1007/s12144-016-9417-3

[ref44] Lau K. S. L. Marsee M. A. (2013). Exploring narcissism, psychopathy, and machiavellianism in youth: examination of associations with antisocial behavior and aggression. J. Child Fam. Stud. 22, 355–367. doi: 10.1007/s10826-012-9586-0

[ref45] LeBreton J. M. Shiverdecker L. K. Grimaldi E. M. (2018). The dark triad and workplace behavior. Annu. Rev. Organ. Psychol. Organ. Behav. 5, 387–414. doi: 10.1146/annurev-orgpsych-032117-104451

[ref46] Lin B. Chen H. (2012). I love to do it or “I can do it?” competing mechanisms in explaining creative deviance. Acad. Manag. 2012:15204. doi: 10.5465/AMBPP.2012.15204abstract

[ref47] Lin B. Mainemelis C. Kark R. (2016). Leaders’ responses to creative deviance: differential effects on subsequent creative deviance and creative performance. Leadersh. Q. 27, 537–556. doi: 10.1016/j.leaqua.2015.09.001

[ref48] Liu H. Chiang J. T. J. Fehr R. Xu M. Wang S. (2017). How do leaders react when treated unfairly? Leader narcissism and self-interested behavior in response to unfair treatment. J. Appl. Psychol. 102, 1590–1599. doi: 10.1037/apl0000237, PMID: 28617000

[ref49] Liu W. Zhang P. Liao J. Hao P. Mao J. (2016). Abusive supervision and employee creativity: the mediating role of psychological safety and organizational identification. Manag. Decis. 54, 130–147. doi: 10.1108/MD-09-2013-0443

[ref50] Liu Q. Zhao Z. Liu Y. Guo Y. He Y. Wang H. (2022). Influence mechanism of employee playfulness personality on employee creative deviance. Front. Psychol. 13:285. doi: 10.3389/fpsyg.2022.821285, PMID: 35645905 PMC9130931

[ref51] Liu F. Zhou K. (2020). Idiosyncratic deals and creative deviance: the mediating role of psychological entitlement. R & D Manag. 51, 433–446. doi: 10.1111/radm.12430

[ref52] Lobbestael J. Baumeister R. F. Fiebig T. Eckel L. A. (2014). The role of grandiose and vulnerable narcissism in self-reported and laboratory aggression and testosterone reactivity. Pers. Individ. Differ. 69, 22–27. doi: 10.1016/j.paid.2014.05.007

[ref53] Mainemelis C. (2010). Stealing fire: creative deviance in the evolution of new ideas. Acad. Manag. Rev. 35, 558–578. doi: 10.5465/amr.35.4.zok558

[ref54] Mao J. Y. Quan J. Li Y. Xiao J. (2021). The differential implications of employee narcissism for radical versus incremental creativity: a self-affirmation perspective. J. Organ. Behav. 42, 933–949. doi: 10.1002/job.2540

[ref55] Martinsen Ø. L. Arnulf J. K. Furnham A. Lang-Ree O. C. (2019). Narcissism and creativity. Pers. Individ. Differ. 142, 166–171. doi: 10.1016/j.paid.2018.09.032

[ref56] Mittal S. Dhar R. L. (2015). Transformational leadership and employee creativity. Manag. Decis. 53, 894–910. doi: 10.1108/MD-07-2014-0464

[ref57] Morrison E. W. Phelps C. C. (1999). Taking charge at work: extrarole efforts to initiate workplace change. Acad. Manag. J. 42, 403–419. doi: 10.5465/257011

[ref58] Nevicka B. Van Vianen A. E. M. De Hoogh A. H. B. Voorn B. C. M. (2018). Narcissistic leaders: an asset or a liability? Leader visibility, follower responses, and group-level absenteeism. J. Appl. Psychol. 103, 703–723. doi: 10.1037/apl0000298, PMID: 29553765

[ref59] Penney L. M. Spector P. E. (2002). Narcissism and counterproductive work behavior: do bigger egos mean bigger problems? Int. J. Sel. Assess. 10, 126–134. doi: 10.1111/1468-2389.00199

[ref01] Podsakoff P. M. MacKenzie S. B. Lee J. Y. Podsakoff N. P. (2003). Common method biases in behavioral research: a critical review of the literature and recommended remedies. J Appl Psychol. 88, 879–903. doi: 10.1037/0021-9010.88.5.879 14516251

[ref60] Qu R. Janssen O. Shi K. (2015). Transformational leadership and follower creativity: the mediating role of follower relational identification and the moderating role of leader creativity expectations. Leadersh. Q. 26, 286–299. doi: 10.1016/j.leaqua.2014.12.004

[ref61] Redmond M. R. Mumford M. D. Teach R. (1993). Putting creativity to work: effects of leader behavior on subordinate creativity. Organ. Behav. Hum. Decis. Process. 55, 120–151. doi: 10.1006/obhd.1993.1027

[ref62] Resick C. J. Whitman D. S. Weingarden S. M. Hiller N. J. (2009). The bright-side and the dark-side of CEO personality: examining core self-evaluations, narcissism, transformational leadership, and strategic influence. J. Appl. Psychol. 94, 1365–1381. doi: 10.1037/a0016238, PMID: 19916649

[ref63] Robinson S. L. Bennett R. J. (1995). A typology of deviant workplace behaviors: a multidimensional scaling study. Acad. Manag. 38, 555–572. doi: 10.5465/256693

[ref64] Rosenthal S. A. Pittinsky T. L. (2006). Narcissistic leadership. Leadersh. Q. 17, 617–633. doi: 10.1016/j.leaqua.2006.10.005

[ref65] Shah M. Sarfraz M. Khawaja K. F. Tariq J. (2020). Does narcissism encourage unethical pro-organizational behavior in the service sector? A case study in Pakistan. Glob. Bus. Organ. Excell. 40, 44–57. doi: 10.1002/joe.22062

[ref66] Shin S. J. Zhou J. (2003). Transformational leadership, conservation, and creativity: evidence from Korea. Acad. Manag. J. 46, 703–714. doi: 10.5465/30040662

[ref67] Shukla J. Kark R. (2020). Now you do it, now you don’t: the mixed blessing of creative deviance as a prosocial behavior. Front. Psychol. 11:313. doi: 10.3389/fpsyg.2020.00313, PMID: 32194478 PMC7066209

[ref68] Smith M. B. Webster B. D. (2018). Narcissus the innovator? The relationship between grandiose narcissism, innovation, and adaptability. Pers. Individ. Differ. 121, 67–73. doi: 10.1016/j.paid.2017.09.018

[ref69] Song C. Lee C. H. (2020). The effect of service workers’ proactive personality on their psychological withdrawal behaviors: a moderating effect of servant leadership. Leadersh. Organ. Dev. J. 41, 653–667. doi: 10.1108/LODJ-04-2019-0149

[ref70] Spurk D. Keller A. C. Hirschi A. (2016). Do bad guys get ahead or fall behind? Relationships of the dark triad of personality with objective and subjective career success. Soc. Psychol. Personal. Sci. 7, 113–121. doi: 10.1177/1948550615609735

[ref71] Tenzer H. Yang P. (2019). Personality, values, or attitudes? Individual-level antecedents to creative deviance. Int. J. Innov. Manag. 23:1950009. doi: 10.1142/s1363919619500099

[ref72] Tett R. P. Burnett D. D. (2003). A personality trait-based interactionist model of job performance. J. Appl. Psychol. 88, 500–517. doi: 10.1037/0021-9010.88.3.500, PMID: 12814298

[ref73] Tett R. P. Toich M. J. Ozkum S. B. (2021). Trait activation theory: a review of the literature and applications to five lines of personality dynamics research. Annu. Rev. Organ. Psychol. Organ. Behav. 8, 199–233. doi: 10.1146/annurev-orgpsych-012420-062228

[ref74] Tierney P. Farmer S. M. (2002). Creative self-efficacy: its potential antecedents and relationship to creative performance. Acad. Manag. J. 45, 1137–1148. doi: 10.5465/3069429

[ref75] Tierney P. Farmer S. M. (2011). Creative self-efficacy development and creative performance over time. J. Appl. Psychol. 96, 277–293. doi: 10.1037/a0020952, PMID: 20954756

[ref76] Vadera A. K. Pratt M. G. Mishra P. (2013). Constructive deviance in organizations: integrating and moving forward. J. Manag. 39, 1221–1276. doi: 10.1177/0149206313475816

[ref77] Wallace H. M. Baumeister R. F. (2002). The performance of narcissists rises and falls with perceived opportunity for glory. J. Pers. Soc. Psychol. 82, 819–834. doi: 10.1037/0022-3514.82.5.819, PMID: 12003480

[ref78] Wang S. Liu Y. Shalley C. E. (2018). Idiosyncratic deals and employee creativity: the mediating role of creative self-efficacy. Hum. Resour. Manag. 57, 1443–1453. doi: 10.1002/hrm.21917

[ref79] Wang C. J. Tsai H. T. Tsai M. T. (2014). Linking transformational leadership and employee creativity in the hospitality industry: the influences of creative role identity, creative self-efficacy, and job complexity. Tour. Manag. 40, 79–89. doi: 10.1016/j.tourman.2013.05.008

[ref80] Zeigler-Hill V. Besser A. (2013). A glimpse behind the mask: facets of narcissism and feelings of self-worth. J. Pers. Assess. 95, 249–260. doi: 10.1080/00223891.2012.717150, PMID: 22946774

[ref81] Zhang H. Ou A. Y. Tsui A. S. Wang H. (2017). CEO humility, narcissism and firm innovation: a paradox perspective on CEO traits. Leadersh. Q. 28, 585–604. doi: 10.1016/j.leaqua.2017.01.003

[ref82] Zheng Y. Huang X. Graham L. Redman T. Hu S. (2020). Deterrence effects: the role of authoritarian leadership in controlling employee workplace deviance. Manag. Organ. Rev. 16, 377–404. doi: 10.1017/mor.2019.50

